# Analysis of recurrent research pathways for assessing and improving effectiveness in life sciences laboratories

**DOI:** 10.1101/2023.01.09.23284360

**Published:** 2023-01-10

**Authors:** E. Andrew Balas, Charmi Patel, Ben Ewing, Nauka Patel, Tiana Curry McCoy, Scott Wise, Yara H Abdelgawad

**Affiliations:** 1 Biomedical Research Innovation Laboratory at Augusta University GA; 2 Clinical Laboratory Science Program at Augusta University GA

**Keywords:** life sciences research, quality improvement, practice variation, companion diagnostics, virus variants, vaccine development

## Abstract

**Background:**

Life sciences research often turns out to be ineffective. Our aim was to develop a method for mapping repetitive research processes, detecting practice variations, and exploring inefficiencies.

**Methods:**

Three samples of R&I projects were used: companion diagnostics of cancer treatments, identification of COVID-19 variants, and COVID-19 vaccine development. Major steps involved: defined starting points, desired end points; measurement of transition times and success rates; exploration of variations, and recommendations for improved efficiency.

**Results:**

Over 50% of CDX developments failed to reach market simultaneously with new drugs. There were significant variations among phases of co-development (Bartlett test P<0.001). Length of time in vaccine development also shows variations (P<0.0001). Similarly, subject participation indicates unexplained variations in trials (Phase I: 489.7 (±461.8); Phase II: 857.3 (±450.1); Phase III: 35402 (±18079).

**Conclusion:**

Analysis of repetitive research processes can highlight inefficiencies and show ways to improve quality and productivity in life sciences.

Life sciences research has produced many landmark discoveries for public health improvement but many research projects turned out to be ineffective. Begley and Prinz estimated that the amount of non-reproducible research is somewhere between 75 to 89%, astoundingly high rates (Begley, 2012, Prinz, 2011). Improving the quality and productivity of scientific research is a paramount societal interest. Part of the difficulty relates to the complexity of the process involved in translating new biologic and technologic developments into routine clinical medicine (Parkinson, 2012).

Scientific research is inherently creative, variable, and unpredictable, making it difficult to help with quality improvement efforts. On the other hand, several important research processes turn out to be repetitive and therefore analyzable for improving performance. In such cases, information can be collected about multiple occurrences of the same or similar processes and analyzed for optimization. The following three examples from life sciences research illustrate the many repetitive processes and the need for improvement:

A growing number of companion diagnostics aims to identify patients that will respond to targeted therapies, thus increasing efficacy and safety of the drug. The pace of progress in linking targeted therapies with appropriately characterized biomarkers and patients is known to be frustratingly slow. The post launch delay is approximately 4.5 years, and it takes from 1.5 to 5 years to offer the test by labs to achieve optimal rate after treatment introduction (Keeling et al, 2020). Failure to obtain the right target for the cancer treatment led to the failure to get the desired results in many drugs clinical trials which has overall impact on initial stages of co-development process (Hu, 2019). Failure to obtain information about different diagnostic metrics affects the evaluation of clinical validity of the assay (Jorgensen, 2018).

Virus variants are coming up quickly and randomly, but we are slow in identifying them. The current process to identify and classify SARS-COV-2 variants is a detailed process that takes up to a month from the time of a positive tests for COVID-19 to the identification and classification of a variant. State health departments prepare and send positive COVID-19 samples to the CDC weekly or biweekly. It then takes about ten days for the CDC or their contracted agencies to perform genetic sequencing and identify variants (CDC, 2021). The US is behind 30 other countries in the amount of sequencing done during the pandemic despite having the equipment and experience to do more (Maxmen, 2021). Much of COVID-19 testing is done in labs that do not have genomic sequencing capabilities, therefore samples are often discarded due to the need for extra storage and labor needed to store samples (Maxmen, 2021).

The rapid spread and devastating consequences of the COVID-19 pandemic have changed the vaccine development processes. In response to the need to expedite development which previously took many years, researchers pushed for adjustments to regulation and processes, such as the decision to begin Phase 1 clinical trials before the completion of animal testing (Nguyen, 2021). The Pfizer and Moderna vaccines’ Phase 1 and preclinical animal trials were initiated in parallel as early as April 2020, with parallel Phases 2 and 3 being run three months later (Kieny, 2021). In early 2020, it was unethical to use human challenge studies for SARS-CoV-2 vaccine development (Kahn, 2020). This made it difficult to determine vaccine efficacy in variant subjects and this has been problematic as the controlled human infection models can accelerate vaccine development (Rahman, 2019).

In response to these challenges, comparative study and reengineering of research processes may help to develop faster research response systems. The purpose of this study was to develop and pilot test the systematic analysis of repetitive research processes and find what appears to be ineffective or ready for improvement.

## Methods

The evaluation method of this study is rooted in data science that involves facts and statistics that are available from large databases or published literature. Analysis of recurring research involves the following steps: take a sample of R&I projects; clearly define starting point and define the desired endpoint; measure transition times and success rates; explore variations and weaknesses; and develop recommendation for improved efficiency.

1. Developing eligibility criteria of a repetitive research process: The first step is the identification of recurring research processes which happen with sufficient frequency to observe and analyze a sample of them. These selected research processes should have clearly identifiable starting and end points.
2. Sampling of R&I projects: After setting the eligibility criteria, the next step is to collect a sufficiently large sample research and development projects. It is desirable to have at least 10–30 randomly selected research and development processes to make statistical analysis sufficiently substantial.
3. Defining starting and desired end point: Such definitions are essential to set the boundaries of analysis and create opportunities for comparing multiple research processes and explore weaknesses or deficiencies. Typically, the process starts with the recognition of the need to launch a research process and ends when completion of the research delivers value that is universally recognizable (e.g., FDA approval of safety and efficacy)
4. Flowcharting the process of research: Visual presentation of either the actual or the ideal research process can be very valuable in identifying weaknesses and discrepancies. In much translational type research, the process includes discovery of the opportunity, preclinical study, clinical study with phase I, II and III clinical trials and finally FDA approval.
  The flowchart should help to describe identifiable start and end times of all phases plus the identifiable intermediate results of each phase. The flow chart for the research process should support the assessment of the entire series of interconnected activities. For example, thin arrows can indicate the flow of the process, and bold arrows can highlight the difference between phases.
5. Collecting data about transition times and success rates: Independent variables include availability of the resources from the previous phase and the dependent variables include time and frequency. The data sources of this study included: FDA.gov, clinicaltrials.gov, PubMed. Google Scholar, CDC, USPTO, and others to extract relevant information. Research and commercial R&D database information about initiatives were also retrieved to study patterns of development and repurposing.
6. Exploring variations and weaknesses: Each or most flowcharted steps were evaluated for their transition time from the beginning to the end and for their success rates. Additionally, overall time spans were also evaluated from the beginning to the completion of the entire research process.
  Subsequently, averages, differences, and standard deviations were calculated between the start of phases and for the overall development time. We used Bartlett’s test for the null hypothesis that all population variances are equal against the alternative that at least two are different.
7. Developing recommendation for improved efficiency: Based on the above-described information collection, unexplained variations and avoidable failure rates can be observed and recommended for improvement. Suggested improvements in future research and development processes should offer higher quality results in a timely manner for better public health outcomes.

## Results

This study applied the above described research pathway assessment methodology and looked at the current routine processes in the (i) development of 14 drug-companion diagnostic test pairs as shown in) Table 1; (ii)development of 6 vaccines for the SARS-COV-2 virus; and (iii) detection and classification of 11 known variants to the SARS-COV-2 virus as of December 2021 (Table 1c). The estimated percent increase in transmission from the original SARS-COV-2 strain to 5 of the known variants is also displayed. The table also shows where and when the variants were first identified; the highest classification the variant received and when it received, and the current classification.

In addition to the table version of the research flow, results of the research process exploration and visualization can also be seen in three flowcharts. The process in the development of companion diagnostics can be seen in Figure 1a. Development of vaccines against Covid-19 are presented on Figure 1b. Finally, the process of variant identification of the SARS-COV-2 virus is shown in Figure 1c.

### Weaknesses of Transition Times

In the development of companion diagnostics, the average development time of the new drug and CDX is 5.12 and 4.72 years, respectively, but the 4.92 years approval gap is remarkable. Life-threatening side effects have also been detected with an average of 45.17% (± 19.98%) in phase 1 trials.

Obviously, timelines for the development of the mRNA-based Pfizer and Moderna vaccines (as seen in Table 1b) were significantly reduced in comparison to viral vector and non-replicating protein vaccinations, with vaccine efficacy seeing a marked improvement. In the last 2 decades, ≥50% (7/14) of drug-CDX co-developments failed to access the market at the same time.

The current process to identify and classify SARS-COV-2 variants is a detailed series of steps that takes up to a month from the time of a positive tests for COVID-19 to the identification and classification of a variant. Apparently, politics and government/state funding also contribute to the slow speed variants are identified.

### Research Practice Variations

In the analysis of research practice variations, there have been significant and unexplained variations between phases of co-development process time of companion diagnostics (Bartlett test P<0.001).

Similarly, there was a large amount of variation when looking at the process of COVID-19 vaccine development. This high level of variation is reflected in the high standard deviations that are seen in Table 1b, Table 2b, and table 3. There was variation in the number of days each phase of the clinical trial between the 6 different COVID-19 vaccines. While the transition times for both the non-replicating viral vector adjuvant and protein subunit matrix M vaccines ranged from 75 to 116 days between trials, the mRNA based Moderna and Pfizer vaccines averaged half that at only fifty-one days.

There was also large variation in participants in the drug trials of the vaccines investigated and between the different phases of drug trials. Table 3 shows the total subject numbers in vaccine trials were relatively similar between the mRNA-based, viral vector, and non-replicating protein vaccinations, with the Sanofi vaccine having the least participants at 6,576 subjects across all three phases. Other vaccines’ subject pools ranged from 30,645 for the Moderna to 62,090 for the Johnson & Johnson.

We also looked at the process of identifying and classifying variants to the SARS-COV-2 virus. Particular attention was given to the frequency of positive Covid-19 sequences submitted by 50 randomly selected countries which can be seen in Figure 2a. The frequency of positive Covid-19 sequences submitted by each of the 50 US states also shows enormous variation) Figure 2a).

Amongst the 50 randomly selected countries there was a high variability of the number of positive COVID-19 samples sequenced with an average 179,491 samples sequenced and an average percent sequenced of 3.28 with a standard deviation of 558600.04 and 3.55 respectively which can be seen in Table 2. Amongst all 50 US states the state with the highest ratio of positive samples sequenced per population was Wyoming with 3.4%. There seems to be a correlation between the population size of a country and the percentage sequenced. Iceland surprisingly had a higher percentage of samples sequenced.

## Discussion

Applications of our systematic research flow analysis methodology uncover many previously unexposed shortcomings. In vaccine development, COVID variant detection, and companion diagnostics development, we found major and largely unexplained variations of research processes. Such variations suggest that some of the excesses represent ineffective or wasteful use of resources like development time, certain level of patient recruitment in clinical trials or inconsistent sample collection for variant detection. The detected, surprisingly high rates of unexplained and unjustified variations in companion diagnostic development, vaccine development research and also in the identification of virus variants demand attention.

Our study defines an original, comprehensive methodology for the recognition of major deficiencies in research processes. Considering the unacceptably high ratio of non-reproducible research studies, our methodology is important for quality and reproducibility improvement in life sciences research. With the emergence of new electronic data sources and the pressing need for more trustworthy science, many research projects can be analyzed with our timely method. The propsed methodology is simple, structured and ready for wider use.

The increasing demand for precision medicine also indicates that the current co-development process needs a new paradigm. We suggest the early integration of drug and diagnostic components, which is crucial for fast and timely global commercialization and FDA approval simultaneously. The co-development process requires better understanding of gene biology of the disease and the drug mechanism of action to generate a strong biomarker hypothesis for prototype assay development and clinical trials. Simultaneously, the specificity of the companion diagnostic is also essential: a) for the true selection of patients who would be most likely to benefit for the specific drug, b) to recognize the patient at risk for the selected therapeutic product, and c) to monitor the response of the drug dosage, effectiveness, and safety of the selected therapy. The typical development of companion diagnostics needs to be contemporaneous.

It is noteworthy that the mRNA vaccine development times were significantly shorter than the protein and viral vector vaccine developments, two of which just gained FDA approval at the end of 2021 with another gaining approval in January 2022. There was a direct connection between mRNA research done over the last two decades to the research done on mRNA vaccines more recently, which drastically reduced production time and led to a record-setting COVID-19 vaccine development time.

In identification of COVID-19 variants, more worldwide cooperation would also benefit the identification process, particularly learning from the success of countries like Iceland and Denmark. The United States, despite having some of the most advanced technology, is behind many other countries regarding the number of samples sequenced. The time it takes from an increase in positive SARS-COV-2 cases to variant classification needs to be decreased. The frequency that sequences are submitted must increase in many but not all states. Future studies could look at how the political climate of a region affects the submission of sequences and the identification of disease variants as well as how governments affect variant research.

## Figures and Tables

**Figure 1a:**
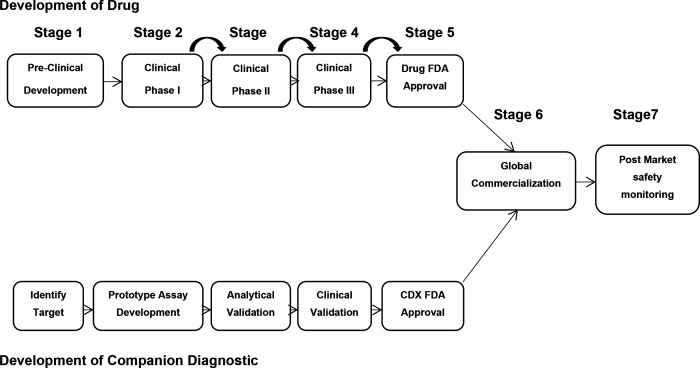
Ideal co-development process of drug and companion diagnostics

**Figure 1b:**
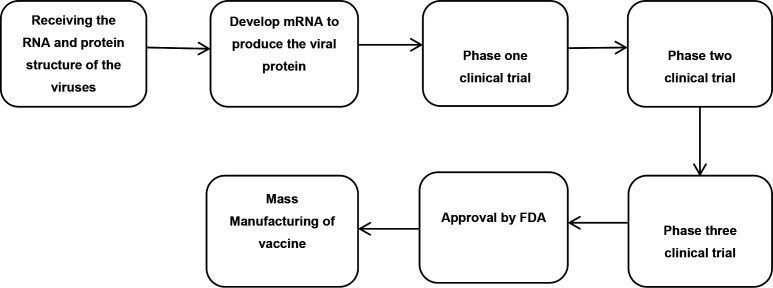
Current Process of Vaccine Development for COVID-19

**Figure 1c:**
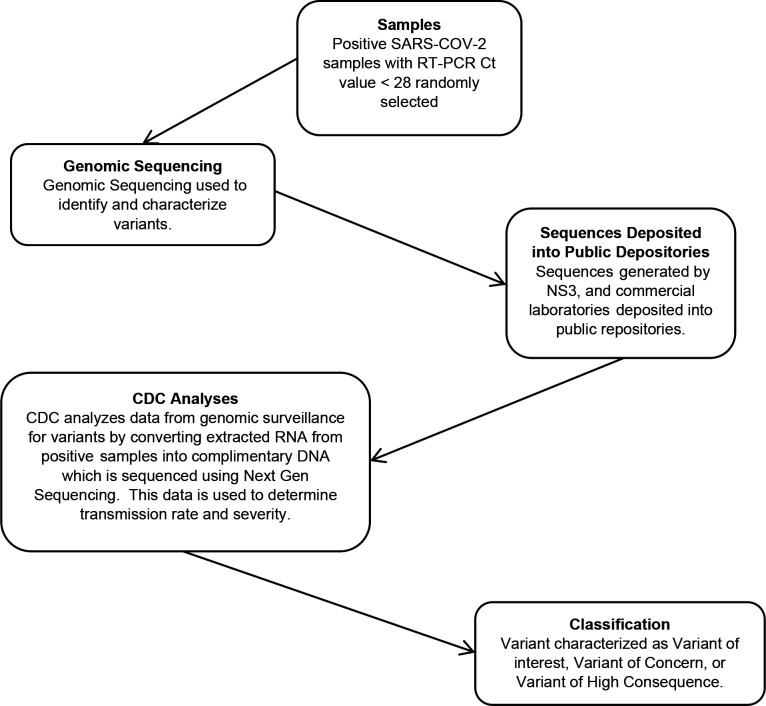
Current process of Identifying and Classifying Variants of SARs-COV-2

**Figure 2a:**
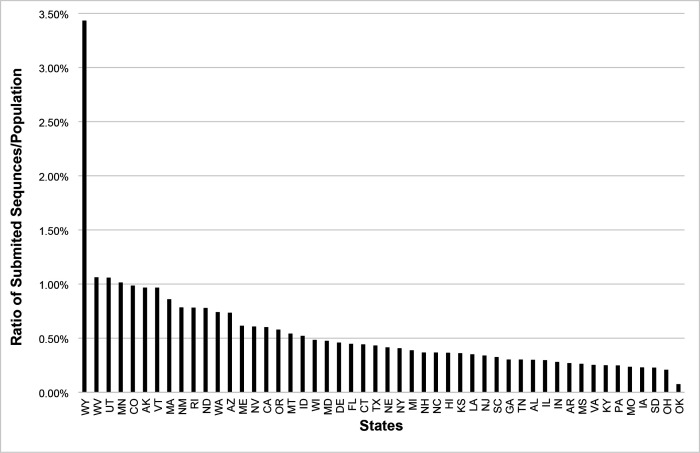
Ratio Sequences/ population of the 50 US States.

**Figure 2b:**
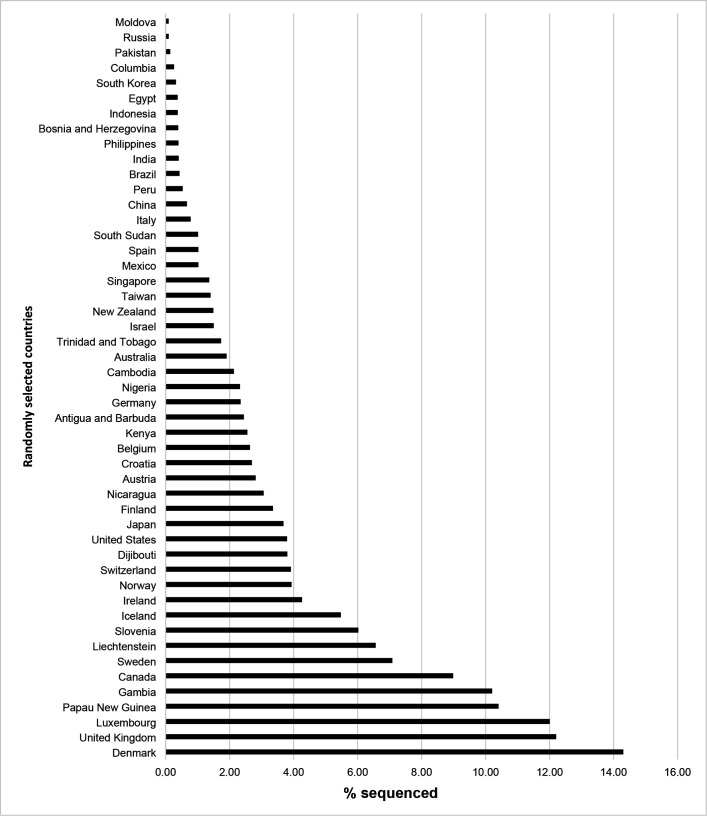
% Sequenced of Positive Covid-19 Samples of 50 Randomly Selected Countries.

**Table 1a:** The development process of analyzed companion diagnostics pairs

Companion Diagnostics	Drugs	Biomarkers	Diseases	Was approved together?	Aproval of CDX	Aproval of drug
Abott Real time IDH1	Tibsovo	IDH1	Acute Myeloid Leukemia	Yes	Jul-18	Jul-18
FoundationOne CDx	Alecensa	ALK rearrangements	Non-small cell lung cancer	No	17-Nov	Dec-15
MRDx BCR-ABL test	Tasigna	Philadelphia Chromosome positive	Chronic Myeloid Leukemia	No	17-Dec	Oct-07
Cobas KRAS mutation test	Vectibix	KRAS mutation Negative	Colorectal cancer	No	May-I5	Sep-06
BRCA analysis CDx	Lynparza	BRCA 1/2, HR+ AND HER2 Negative	Breast Cancer	Yes	Jan-18	Jan-18
Myriad mychoice CDx	Zejula	BRCA1/2	Ovarian Cancer	No	Oct-19	Mar-17
BRCA analysis CDx	Lynparza	germline BRCA	Pancreatic Cancer	Yes	Dec-l9	Dec-19
FoundationOne CDx	Erleada	mCRPC with BRCA1/2	Prostate Cancer	No	Nov-17	Feb-18
HER2 FISH pharmDx	Herceptin (ENHERTU)	HER2	Gastric or Gastroesophageal Cancer	N/A	Oct-10	Jan-21
Vysis CLL FISH Probe	Venetoclax	LSI TP53	B-cell chronic limphocytic leukemia	Yes	Apr-I6	Apr-16
THXID BRAF assay	Tafinlar	BRAF	Melanoma	Yes	May-13	May-13
FoundationOne CDx	Pemazyre	FGFR2	Cholangiocarcinoma	N/A	Nov- I7	Apr-20
Therascreen FGFR RGQ RT-PCR test	Balversa	FGFR3	Urothelial (Bladder) cancer	Yes	Apr-I9	Apr-19
VENTANA MMR RXDX Panel	Jemperli	DMMR	Endometrial Carcinoma	Yes	Apr-21	Apr-21

**Table 1b:** Length of time comparisons of phases in vaccine development projects (days).

Development of Vaccine	Diff. Preclinical-Phase 1	Diff. Phase I-Phase II	Diff. Phase II-Phase III	Diff. Phase III-FDA approval
BioNTech/Pfizer	30.00	30.00	30.00	30.00
Moderna	22.00	73.00	58.00	0.00
AstraZeneca	26.00	28.00	97.00	122.00
Janssen (Johnson & Johnson)	105.00	0.00	66.00	156.00
Novavax	25.00	0.00	-	-
GSK/Sanofi	21.00	716.00	95.00	-
Average (±SD)	38.17 (±32.90)	141.2 (±282.9)	69.2 (±27.9)	77.00 (±73.9)

**Table 1c:** COVID-19 variant transmissibility, identification, and classification

Variants	Where First Identified	Earliest Documented Samples	Highest Classification	Current Variant Classification	Estimated Transmissibility increase (%)
Alpha B.1.1.7	United Kingdom	Sep-20	VOC: December 29, 2020	VBM	29
Beta B.1.351	South Africa	May-20	VOC: December 29, 2020	VBM	25
Gamma P.1	Japan/Brazil	Nov-20	VOC: December 29, 2020	VBM	38
Delta B.1.617.2	India	Oct-20	VOC	VOC	97
Kappa B.1.617.1	India	Oct-20	VOI: May 7, 2021	VBM	48
Mean					47.4
Standard Deviation					29.11
Eta B.1.525	Nigeria	Dec-20	VOI: February 26,2021	VBM	
Omicron B.1.1.529	Multiple countries	Nov-21	VOC: November 26,2021	VOC	
Iota B.1.526	New York, U.S.	Nov-20	VOI: February 26,2021	VBM	
Mu B.1.621	Columbia	Jan-21	VBM: September 21, 2021	VBM	
Epsilon B.1.427 and B.1.429	California, U.S	Sep-20	VOC: March 19,2021	VBM	
Zeta P.2	Brazil	Jan-21	VOI: February 26, 2021	VBM	

**Table 2a:** Difference between phases of co-development process and total development time of Companion Diagnostics

Difference between	Average days (±SD)
Start of Phase I & II	951.82 (±977.29)
Start of Phase II & III	552.50 (±457.21)
Start of Phase III & FDA approval	771.43 (±627.9)
Drug patented & FDA approval	394.62 (±263.21)
Approval lag between CDX & Drug	822.50 (±1297.33)
Duration of the CDX development	1718.08 (±829.92)
Duration of the Drug Development	1863.68 (±1375.92)

**Table 2b:** Group specific average transition times (start of preclinical testing to FDA approval)

Vaccine	Average	Standard Deviation
mRNA	207	123.04
Non-replicating viral vector	300	38.18
Protein subunit + matrix M adjuvant	465.50	232.64

**Table 3:** Subject participation in COVID-19 vaccine clinical trials

Development of Vaccine	Phase 1 subjects	Phase 2 subjects	Phase 3 subjects
BioNTech/Pfizer	200	200	44,000
Moderna	45	600	30,000
AstraZeneca	1,077	1,077	40,000
Janssen (Johnson& Johnson)	1,045	1,045	60,000
Novavax	131	1,500	33,000
GSK/Sanofi	440	722	5,414
**Average (±SD)**	**489.7 (±461.8)**	**857.3 (±450.1)**	**35402 (±18079)**

## Abbreviations:

R&I: Research and Innovation
COVID-19: coronavirus disease of 2019
SARS-COV-2: Severe acute respiratory syndrome coronavirus 2
PubMed: Publisher MEDLINE
USPTO: United States Patent and Trademark Office
R&D: Research and Development
ACE: Angiotensin Converting Enzyme
CDX: Companion Diagnostics

## References

[1] Beeler J. Integrating companion diagnostic assays into drug development: addressing the challenges from the diagnostic perspective. Drug Development Research. 2013 Mar;74(2):148–54.

[2] Begley CG, Ellis LM. Raise standards for preclinical cancer research. Nature. 2012 Mar;483(7391):531–3. 22460880 10.1038/483531a

[3] COVID-19 Vaccination. Centers for Disease Control and Prevention (2021). https://www.cdc.gov/coronavirus/2019-ncov/vaccines/distributing/steps-ensure-safety.html. 34009769

[4] Hu C, Dignam JJ. Biomarker-driven oncology clinical trials: Key design elements, types, features, and practical considerations. JCO Precision Oncology. 2019 Oct;1:1–2. 10.1200/PO.19.00086 PMC7446374 32923854

[5] Jorgensen JT, Hersom M. Clinical and regulatory aspects of companion diagnostic development in oncology. Clinical Pharmacology & Therapeutics. 2018 Jun;103(6):999–1008. 29197081 10.1002/cpt.955

[6] Kahn JP, Henry LM, Mastroianni AC, Chen WH, Macklin R. For now, it’s unethical to use human challenge studies for SARS-CoV-2 vaccine development. Proceedings of the National Academy of Sciences. 2020 Nov 17;117(46):28538–42. 10.1073/pnas.2021189117 PMC7682548 33122444

[7] Keeling P, Clark J, Finucane S. Challenges in the clinical implementation of precision medicine companion diagnostics. Expert review of molecular diagnostics. 2020 Jun 2;20(6):593–9. 32336167 10.1080/14737159.2020.1757436

[8] Kieny MP. Accelerating vaccine trials. Bull World Health Organ. 2021;99:482–3. 34248219 10.2471/BLT.21.020721 PMC8243025

[9] Maxmen A. Why US coronavirus tracking can’t keep up with concerning variants. Nature. 2021:336–7. 10.1038/d41586-021-00908-0 33828280

[10] Nguyen LC, Bakerlee CW, McKelvey TG, Rose SM, Norman AJ, Joseph N, Manheim D, McLaren MR, Jiang S, Barnes CF, Kinniment M. Evaluating use cases for human challenge trials in accelerating SARS-CoV-2 vaccine development. Clinical Infectious Diseases. 2021 Feb 15;72(4):710–5. 32628748 10.1093/cid/ciaa935 PMC7454474

[11] Parkinson DR, Johnson BE, Sledge GW. Making personalized cancer medicine a reality: challenges and opportunities in the development of biomarkers and companion diagnostics. Clinical Cancer Research. 2012 Feb 1;18(3):619–24. 22298894 10.1158/1078-0432.CCR-11-2017

[12] Prinz F, Schlange T, Asadullah K. Believe it or not: how much can we rely on published data on potential drug targets?. Nature reviews Drug discovery. 2011 Sep;10(9):712–712. 10.1038/nrd3439-c1 21892149

[13] Rahman MA, Robert-Guroff M. Accelerating HIV vaccine development using non-human primate models. Expert Review of Vaccines. 2019 Jan 2;18(1):61–73. 30526159 10.1080/14760584.2019.1557521

